# The fundus slit lamp

**DOI:** 10.1186/s40064-015-0838-5

**Published:** 2015-02-03

**Authors:** Marcus-Matthias Gellrich

**Affiliations:** Ophthalmological practice, Ziegeleiweg 10, 25548 Kellinghusen, Germany

**Keywords:** Fundus imaging, Video slit lamp, Biomicroscopy, Videography, Flicker test, Mosaic function, Retinal surface contour, Converging lens, Aperture

## Abstract

Fundus biomicroscopy with the slit lamp as it is practiced widely nowadays was not established until the 1980-es with the introduction of the Volk lenses +90 and +60D. Thereafter little progress has been made in retinal imaging with the slit lamp. It is the aim of this paper to fully exploit the potential of a video slit lamp for fundus documentation by using easily accessible additions.

Suitable still images are easily retrieved from videorecordings of slit lamp examinations. The effects of changements in the slit lamp itself (slit beam and apertures) and its examination equipment (converging lenses from +40 to +90D) on quality and spectrum of fundus images are demonstrated. Imaging software is applied for reconstruction of larger fundus areas in a mosaic pattern (Hugin®) and to perform the flicker test in order to visualize changes in the same fundus area at different points of time (Power Point®).

The three lenses +90/+60/+40D are a good choice for imaging the whole spectrum of retinal diseases. Displacement of the oblique slit light can be used to assess changes in the surface profile of the inner retina which occurs e.g. in macular holes or pigment epithelial detachment. The mosaic function in its easiest form (one strip macula adapted to one strip with the optic disc) provides an overview of the posterior pole comparable to a fundus camera’s image. A reconstruction of larger fundus areas is feasible for imaging in vitreoretinal surgery or occlusive vessel disease. The flicker test is a fine tool for monitoring progressive glaucoma by changes in the optic disc, and it is also a valuable diagnostic tool in macular disease.

Nearly all retinal diseases can be imaged with the slit lamp - irrespective whether they affect the posterior pole, mainly the optic nerve or the macula, the whole retina or only its periphery. Even a basic fundus controlled perimetry is possible. Therefore fundus videography with the slit lamp is a worthwhile approach especially for the many ophthalmologists without access to the most recent diagnostic equipment or a professional photographer at hand.

**Electronic supplementary material:**

The online version of this article (doi:10.1186/s40064-015-0838-5) contains supplementary material, which is available to authorized users.

## Introduction

Biomicroscopy of the fundus with the slit lamp is ultimately the result of melding the two most important examination techniques in clinical ophthalmology, namely biomicroscopy with the slit lamp and fundoscopy. Historically speaking, fundoscopy is the older examination technique, dating back to 1851 (Helmholtz 1909), while the slit lamp was introduced by Allvar Gullstrand in 1911 (Gullstrand 1911). But it lasted several more decades until with the introduction of the +90 and +60D Volk lenses in the 1980-ies clinical biomicroscopy of the fundus gained its present form (Lundberg 1985; Volk 1986). This is even more astonishing as two important preconditions had been avaible since the 1950-ies: the theoretical concept for fundus biomcroscopy through a high power convex lens (El Bayadi 1953; El Bayadi 1965) and a slit lamp which allowed a nearly parallel beam between observation and illumination (Littmann 1950a).

Till the introduction of the Volk lenses the concept of concave lenses for fundus observation with the slit lamp had been pursued (Littmann 1950b), which had been introduced by Leonhard Koeppe with a contact lens (Koeppe 1918) and Karl Hruby through a hand held lens (Hruby 1941; Hruby 1950). From that earlier period the Goldmann three mirror contact lens has survived which is still a reliable clinical tool for examination of the macula and the fundus periphery (Goldmann 1949). Its disadvantages are the small area of visible retina, the need of touching the cornea and dilating the pupils.

Furthermore the traditional concept of the slit lamp for anterior segment biomicroscopy seems so strong (Berliner 1949; Holland 2004; Koeppe 1922; Koppenhöfer 2011) that until now its manufacturers have made no changes in the construction which supports posterior segment biomicroscopy specifically (Meesmann 1927; Shulpina 1966; Thiel 1930; Vogt 1930, 1931, 1942). Their brochures - if at all - contain very few fundus material (Müller & Wagner 2001) and even on the free book market until recently no larger collection of slit lamp findings of the fundus with the present methods was available (Hruby 1950; Gellrich 2011a).

It is true that many new tools have entered the scene for fundus documentation (Flittiger 2012), but it is also true, that contemporary ophthalmologists find themselves spending increasing time at the slit lamp on posterior segment biomicroscopy. We therefore see the need to demonstrate the slit lamp’s capacities for posterior segment imaging, by evaluating the instrument itself (beam, apertures) and its examinations tools (fundus lenses), but also the potential advantages of modern video imaging and processing (Saine & Tyler 2002; Tyler et al. 2003; Wolffsohn 2008). Based on these techniques in this article the wide and in important parts unknown spectrum of fundus documentation with the slit lamp will be presented (Gellrich 2009a; Gellrich 2013a).

## Material and methods

A Zeiss SL 105 slit lamp was used with an inbuilt CCD video camera (Panasonic, GP-KS 162 HDE with 752×582 pixels). Improvements for slit lamp-imaging were investigated in two different fields: (1) the slit lamp and examination equipment, and (2) the digital image path.

1. To examine the posterior segment, the Volk + 90D, +60D, +40D and other lens powers (+55D, +20D) were used. Effects of different lens powers on examination conditions (position of the slit lamp), visible field size and magnification were evaluated.

**Figure 1:**
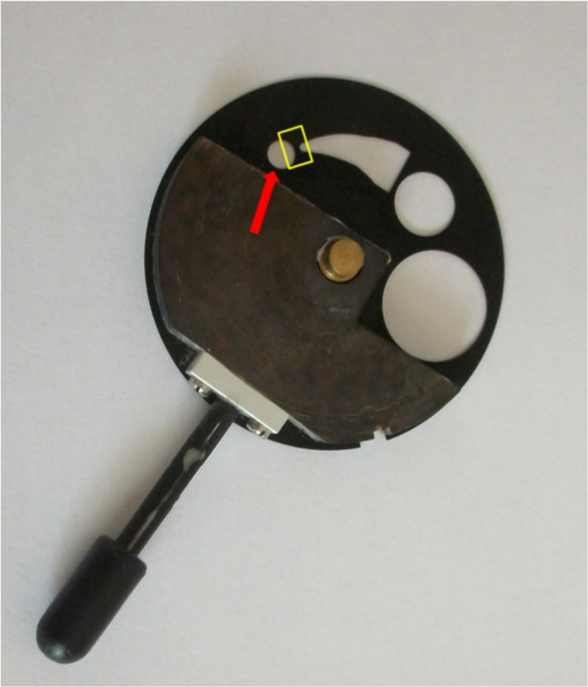
**Aperture plate from Zeiss SL 105 slit lamp showing crescent-shaped and circular apertures.** The red arrow points to the 3.5 mm circular aperture (which is projected onto the fundus in Figure 3). If this plate is rotated in an intermediate position, with the aid of the second aperture for the width of the slit beam (see yellow rectangle) two halfcircles visible in Figure 5 can be projected onto the fundus.

The shape of the projected “slit” in the slit lamp is determined by the superposition of two aperture systems: 4 circular apertures and a crescent-shaped aperture located on a rotatable aperture plate for its basic shape **(**Zeiss type SL 105, Figure 1) and another “curtain-like” aperture which determines the width of the slit. Different settings of these aperture systems were tested in their potential use for slit lamp videography of the fundus.

(2) The slit lamp examination was recorded with an HDD recorder (Panasonic DMR-EX95V, capture rate 25 frames/second). Usually after the examination single frames within the video sequence were identified on which contrast and brightness were sufficient to identify the small arterioles on the fundus and which could serve as a basis for diagnosis and follow up. They were stored as still images on a CF-card (Gellrich 2009a; Gellrich 2009b; Gellrich 2011b) and used for further processing. PowerPoint® was used to perform geometric operations (tilting, cutting, adapting pictures) in order to perform the flicker test. The basic idea is that changement becomes evident by movement of structure when flicking between adjacent foils (Berger et al. 2000; Gellrich 2012). Earlier on we also used these functions to create retinal mosaics (Gellrich 2009c; Gellrich 2009d), but this can be performed at a higher standard using the program Hugin® for image processing (Gellrich 2014a), which allows to “stitch” these mosaics together digitally. PowerPoint® was also used to enhance the image quality by changes in contrast and brightness.

Although it would have been easy to rectify the captured images digitally we left them inverted. This facilitates their use in daily clinical work for direct comparison with the view through the slit lamp oculars.

## Results

1. Slit lamp and examination equipment

### Basic settings

**Figure 2:**
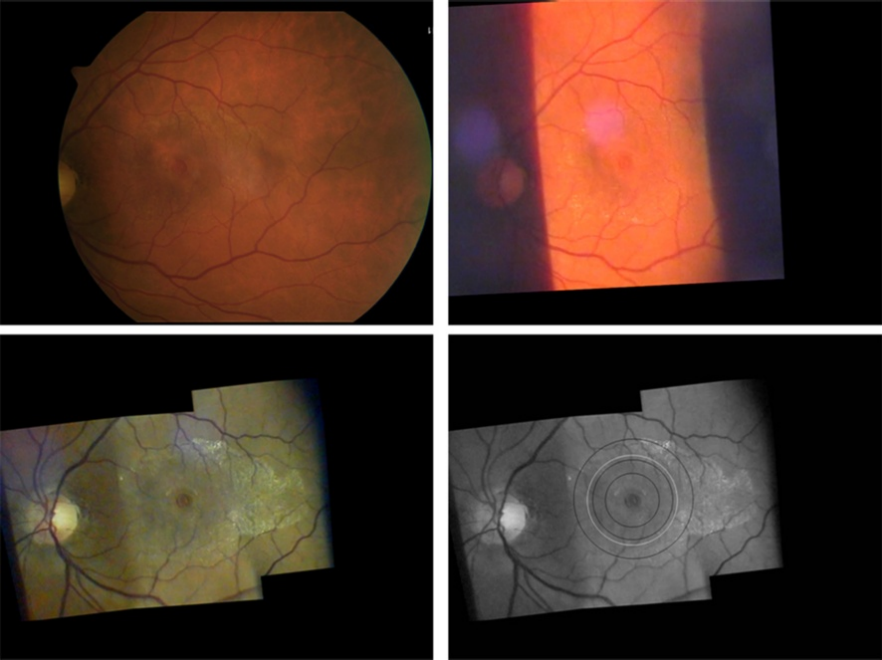
**Comparison between fundus photography and slit lamp videography for a right eye with macular hole and epiretinal membrane (here at same magnification, but original images captured at the settings given below - all figures in this article inverted as seen through the slit lamp).** Top left: 50° posterior pole image - taken with a fundus camera (Zeiss, FF 450, dilated pupil, image inverted for comparison with slit lamp imaging).- Top right: Attempt to capture the pathological process in only one video image taken with the slit lamp (+90D lens, 12x magnification, slit wide open, white light). This image suffers from poor contrast. Reflexes which often disturb slit lamp fundus images are minor in this case. - Bottom left: Mosaic arrangement of 3 video images taken with the slit lamp (+90D lens, 20x magnification, green light) showing the clinically relevant area within the temporal vessel arcade. Single images were arranged with the program Hugin® and further processed with Power Point®. Disease-related features (macular hole, epiretinal membrane) stand out at greater contrast than in the classic fundus photograph top left. - Bottom right: showing the fundus area illuminated by projecting a 3.5 mm circular aperture on the fundus through different fundus lenses (from outer to inner black ring - the higher the lens power, the larger the illuminated fundus area - for comparison see Figure 3). By applying magnification factors given for the respective lenses in a schematic eye (see text) the ring diameters are 4.7 mm (+90D lens), 3.1 mm (+60D lens) and 2.1 mm (+40D lens) and their relation is 2.25 : 1.5 : 1.0. The white ring indicates a circle of 3.5 mm diameter on the retina.

While in traditional fundus photography largely white light is applied to the fundus we find that green light gives better contrast to many fundus structures as epiretinal membranes, vessels and the fovea (Figure 2). If those settings which aim to capture a large fundus area in one picture are applied from fundus photography to slit lamp videography the results will be disappointing with degradation of the image: If image resolution is an important point slit lamp magnifications less than 12x (Gellrich 2009b) and lenses with higher power than the +90D lens (Volk 1986; Lee 1990) should not be used. An exception is if panretinal mosaics are to be created.

For all forms of fundus examination the separation of the beam paths for observation and illumination is an important precondition (Henker 1920; Kaschke et al. 2014). As a rule for the start this can be achieved by using a slit width less than half the pupil size (see Figure 2 bottom left) instead of using a broad slit beam (as is still recommended in Volk’s patent declaration (Volk 1986) - see Figure 2 top right). In addition slight adjustments in the position of the examination lens and the direction of the patient’s gaze should be used by the examiner to “navigate past” disturbing reflexes which is particularly important in imaging of the macula. Holding to these rules will result in image qualities similar to that known from (at least non-mydriatic) fundus photography.

### Converging lenses

The stronger the fundus lens that is used, the shorter its focal distance and the less drawback of the slit lamp (in comparison to the conditions of anterior segment examination) is necessary to be able to observe the real fundus image in the focal plane (approx. 10 cm in front of the slit lamp objective). While the +90D lens requires only 3 cm additional distance between the patient’s eye and the slit lamp’s objective, the +60D lens needs a bit more than 4 cm, and with the + 40D lens approximately 6 cm drawback is necessary. These demands exceed the movements allowed by the dimensions of the slit lamps now on the market, but can be fulfilled if the patient is asked to pull his chin and forehead back a little (approx 3 cm). Once the clinician becomes familiar with these initially unusual examination settings, it is little trouble to use even a + 20D lens, thereby lengthening the examination distance by over 10 cm (in comparison to anterior segment biomicroscopy), but requiring a very small angle between illumination and observation. (One must keep in mind, however, that the intensity of light on the exposed fundus area increases with decreasing power of the fundus lens).

**Figure 3:**
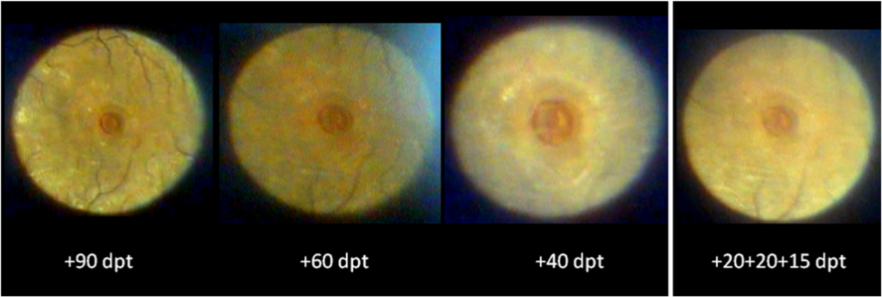
**(same eye as in Figure** **2** **).** View through the slit lamp microscope if a 3.5 mm circular aperture is projected onto the central fundus through lenses with the power +90, +60, +40, +55D (see Figure 4). The higher the power of the examination lens, the less the magnification of the image visible in the slit lamp biomicroscope (also compare to Figure 2, bottom right, for the size of the illuminated fundus area).

It is important to note that the illuminated field’s size visible in the slit lamp oculars reflects the shape and size of the aperture chosen and that it enlarges with the degree of slit lamp magnification, but not with the power of the converging lens. The power of the fundus lens - based on direct proportionality - determines the magnification of the fundus projection of the given illuminated aperture field (Figure 3), namely 1/0.75 for a +90D lens, 1/1.15 for a +60D lens and 1/1.67 for a +40D lens (0.75, 1.15 and 1.67 are the – inversely related - magnification factors given by the manufacturers which are calculated for an emmetropic schematic eye) (Gellrich 2014a). If we apply these values when projecting the 3.5 mm circular aperture (Figure 1, red arrow) on the fundus , we should achieve an illuminated circle of 4.7 mm diameter on the retina if we use a + 90D lens, and 2.1 mm diameter if a +40D lens is used (see Figure 2, bottom right).

It makes sense to use a lens collection (+90/+60/+40D) offering a magnification factor of 1.5 between single fundus lenses - this is the order we know as fixed steps in slit lamp magnifications (in our case 5x, 8x, 12x, 20x, 32x). Additional lenses, however, like the +78D or a lens with 1.0 fundus magnification (as is the case in the central optic of the Goldmann three-mirror lens) fall “in between the steps” and do not offer really new perspectives for clinical use.

**Figure 4:**
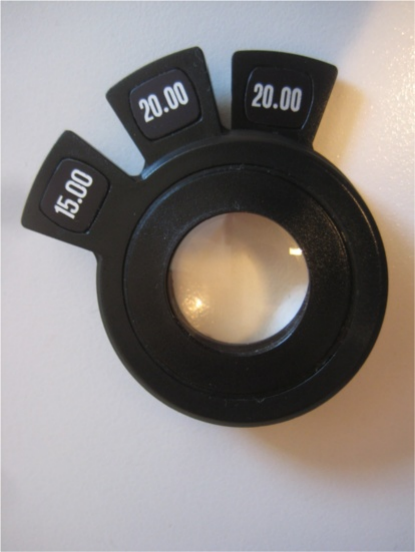
**“Lens sandwich” of altogether +55D power (+20/+20/+15D from lens trial set).** The quality of this “lens sandwich” suffices for adequate diagnosis of the disease condition despite its obvious suboptimal optical properties (in comparison to a single aspheric lens made from glass) - see also Figure 3 (+55D is the power of the original el Bayadi lens (El Bayadi 1953)).

The enormous importance of the power of the converging lens over other lens parameters is underlined by the following observation: We can build a “sandwich” with three lenses from the lens trial set, e.g. +20D/+20D/+15D (Figure 4) forming an overall power of +55D (which is the power of the original Bayadi lens (El Bayadi 1953)). This “composite +55D lens” - despite its poor material quality and little optic refinement - can be employed successfully for fundus biomicroscopy with the slit lamp (Figure 3).

This little experiment - considering also the further ray path through a crystalline lens with opacities and astigmatism - casts doubt on the specific need for a “high end” geometry fundus lens. An aspheric lens design in order to correct for spherical aberrations is particularly required for the more peripheral parts of the examination lens. Near the optic axis, however, spherical aberration plays a minor role (Atchinson & Smith 2002). Fundus examination at the slit lamp mostly happens at slit dimensions less than 5 mm wide and 10 mm high. This fits easily in the diameter of the small +90D lens (approx. 20 mm) which questions the need to introduce “super field “or” ultra wide field” fundus lenses for the slit lamp. Furthermore larger diameters hamper the handling of the lenses close to the patient’s eye (Volk 1986). Also from our experience in clinical work we did not find any advantages of additional features like “digital lenses” specifically designed for digital imaging (Gellrich 2014a).

### Apertures

Among the four possible circular apertures in the Zeiss slit lamp (14 mm, 8 mm, 3.5 mm, 0.3 mm) only that with 3.5 mm diameter can be projected entirely to sufficiently illuminate the fundus, thus providing a good view of the peripapillary or macular area (when used with a +90 or +60D lens at a minimum magnification of 12x - see Figure 3).

**Figure 5:**
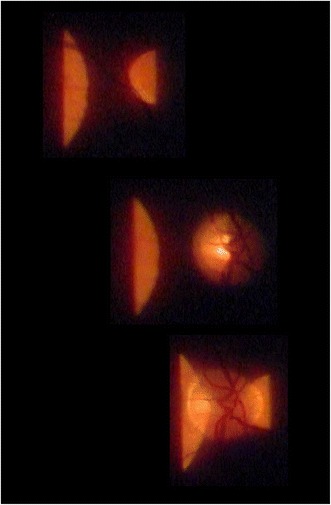
**Aperture segments (see Figure** **1** **- yellow rectangle) from the slit lamp projected through an accessory lens (+90D, 20x magnification) onto the fundus.** This setting may be used for macular examination with less patient’s blinding, steering of the patient’s view and fundus perimetry.- top: the patient sees both semi-circles, − mid: the small semi-circle (within the blind spot) is invisible to the patient,- bottom: inner parts of both semi-circles are hidden for the patient.

Other substructures of the apertures (as known from direct ophthalmoscopy) are not implemented in slit lamps. However, by using an intermediate position between 3.5 mm circular and crescent-shaped aperture and setting the slit width at about 3 mm (Figure 1, yellow rectangle) one can project a “structured” aperture on the fundus (Figure 5). The patient can be asked to look at the dark zone between the two semi-circles (more comfortable, as it is less blinding), and also to fixate on the middle of the small or lower edge of the big semicircle, thus enabling his gaze to be guided during fundoscopy.

A kind of “controlled perimetry of the fundus” is also possible, e.g., the small semi-circle will disappear when projected onto the optic disc (see Figure 5). Since the projection of the Goldmann V-stimulus on the fundus is much smaller than the “small semi-circle” this method can be applied as a broad assessment only of e.g. hemianopias. For the examiner, however, the illuminated area, appears at a uniform level of brightness, which can be modulated by the slit lamp potentiometer. Outside this area the non illuminated fundus structures are practically invisible, which hampers orientation on the retina.

The sharp contrast between directly illuminated and non-illuminated parts of the retina is particulary favourable for the visualization of optic disc drusen by their light-conduction when brightly illuminating only a small part of the optic disc. Sometimes (with a narrow oblique slit light) even the extent of pigment epithelial detachment can be delineated by this method.

**Figure 6:**
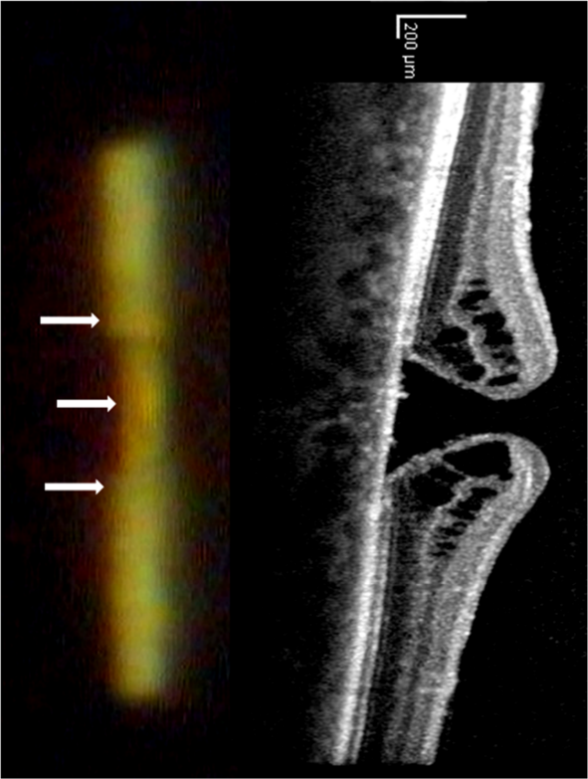
**(same eye as in Figure** **2** **and** **3** **).** Surface analysis of the retina with the slit lamp: Projection of narrow slit beam through a fundoscopic lens (+40D/20x) at a 5° angle temporal to the direction of observation. In the area of the macular hole, the slit light is displaced away from the incident ray (white arrows), indicating a fundus depression. In the end a slit lamp’s “en face” view contains information about the surface contour of the retina by applying the well-known technique of the optic section. On the right the same area is analyzed by a typical vertical OCT section.

The surface structure of the fundus can be evaluated further by a slightly oblique slit beam (e.g. 5° between illumination and observation - see Figure 6). Running over a fundus depression like a macular hole the narrowed slit beam will be deflected away from the side of the slit beam’s arm (this is as seen through the oculars and as shown in this article, but not on the real upright fundus, of course). On the contrary, a fundus elevation (like a tumour, pigment epithelial detachment or large drusen) will deflect the slit beam towards the side of the slit beam’s arm. In both cases the deflection increases with the angle between observation and illumination and with the niveau difference at the fundus. To make this principle as effective as the optic section in anterior segment biomicroscopy a “surround illumination” for easier orientation on the fundus would be an asset.

(2) Modern imaging technology

### Image capturing

Capturing fundus examination in a videosequence - in our eyes - is a prerequisite for satisfying fundus imaging with the slit lamp: While the patient’s fidgeting and blinking during the examination makes real-time photography difficult, one can also obtain images from little children (usually from 4 years onwards) with this method (Szirth et al. 1985). Since the examination and acquisition of useful images may be carried out separately in time and place an assistant can be trained to identify in a videosequence sufficiently sharp still images of for instance the optic nerve and macula, and to store them for further processing (Gellrich 2011b).

### Image processing

All modern techniques of fundus evaluation (digital photography, OCT, HRT, Optomap, autofluorescence, RTA) largely depend on the application of imaging software. Several items are particularly important for fundus imaging with the slit lamp, for example

**Figure 7:**
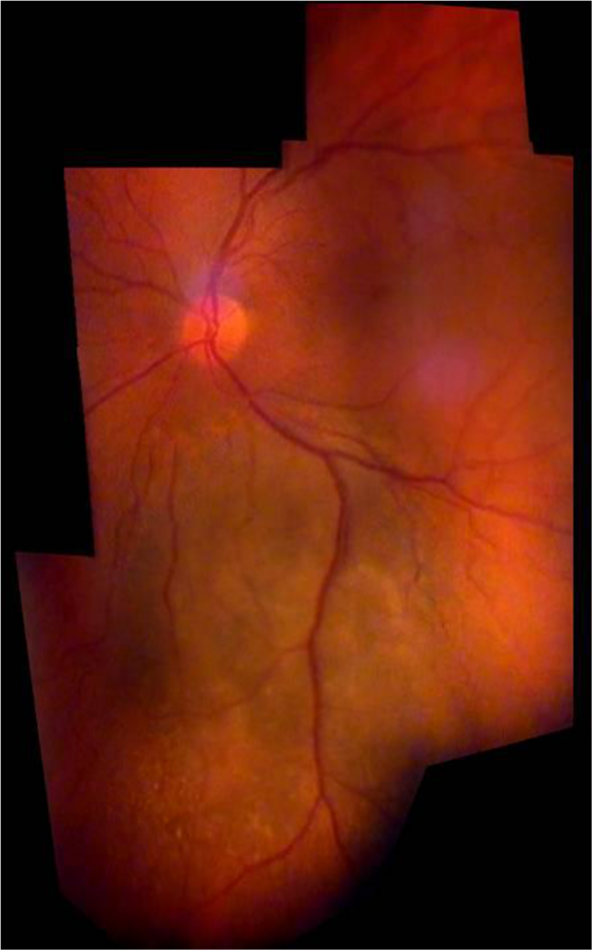
**Choroidal melanoma in a right eye.** Fundus overview created by Hugin® from 6 video images through the slit lamp (+90D lens, 12x magnification).

**Figure 8:**
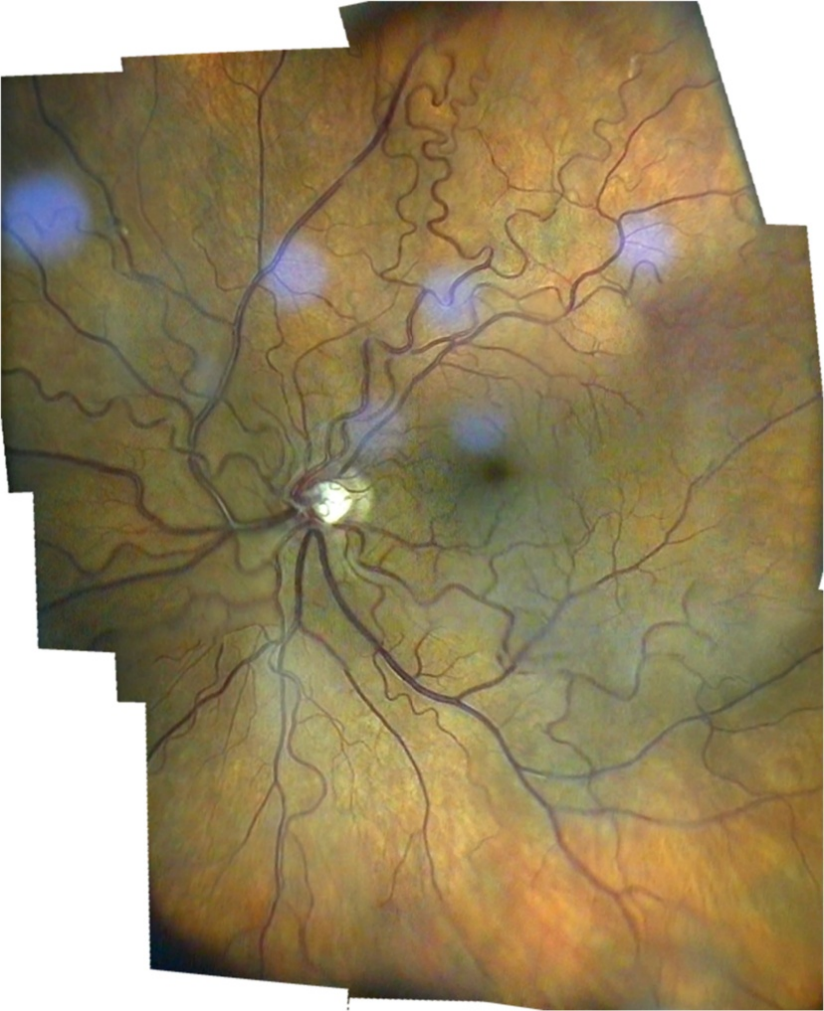
**Fundus mosaic of a right eye with increased vessel tortuosity.** Using Hugin®, 12 video images (slit lamp with +90D lens, 20x magnification, green light) were stitched together digitally (taken from Gellrich, M-M, the slit lamp - applications for biomicroscopy and videography, Springer 2014 (Gellrich 2014a)).

**Figure 9:**
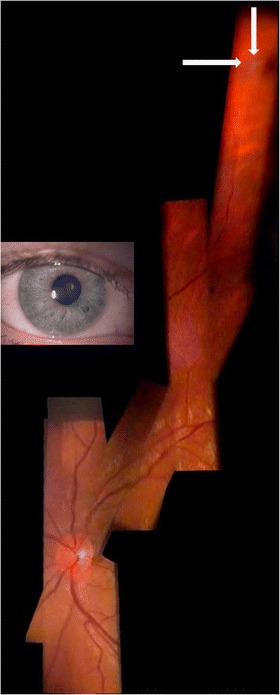
**Fundus of a right eye with peripheral pre-retinal snowballs (white arrows) and adjacent shadow on fundus in a patient with sarcoidosis (slit lamp through narrow pupil as shown in inset, +90D lens/12x magnification).** Composite created using Hugin® and 6 video images.

**Figure 10:**
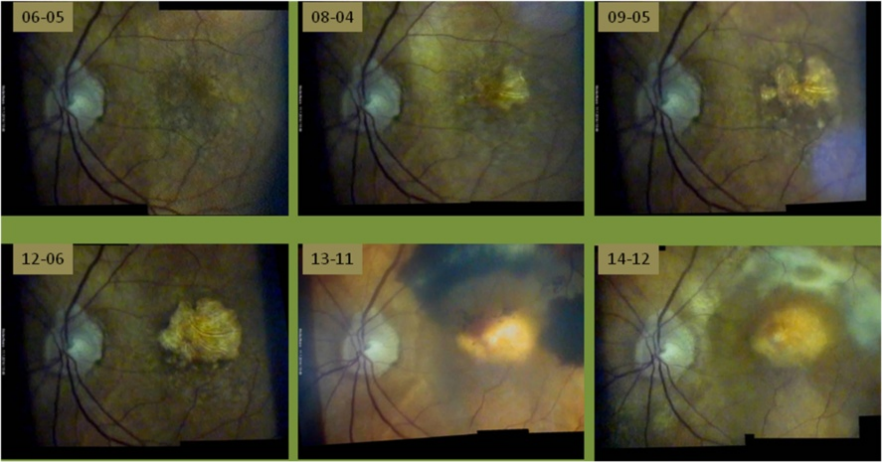
**Slow progression of dry macular degeneration, sudden subretinal hemorrhage after 7½ years and subsequent scarring - documented with the slit lamp using a +90D lens at 20× magnification (dates given yy-mm-dd).** Despite initiation of VEGF-inhibitor treatment (bottom, middle) soon afterwards massive subretinal bleeding occurred. Each picture composite is created from two individual video images by the program Hugin®. Changes between individual images are quite obvious, but alignment with PowerPoint® enables accurate comparison by flicking between consecutive foils. In this case all clinical decisions in 8½ years follow up can be adequately based on the slit lamp documentation.
- Brightness, contrast
  The colour spectrum can be easily altered if the image is imported into PowerPoint® or Picture Manager® (both included in Microsoft Office®) - see Figure 2 bottom right. In our experience many fundus images benefit from a moderate increase in contrast (up to 20%) and a slight reduction in brightness (10%) - see Figures 7, 8, 10, 11.

- the mosaic function
  As its name tells us, only a part of the fundus can be captured in one image with the slit lamp. There are programs available (such as Hugin®) that enable us to geometrically reconstruct a larger fundus area by creating a mosaic. For effective clinical use we advise to make the patient gaze in consecutive directions in a structured manner (Gellrich 2009b). In general this is done in the same way as during a normal clinical fundus examination with the slit lamp. Sufficient (usually approximately 2°) overlap between adjacent fundus segments is necessary, however, so that they can later be stitched together digitally (Figures 7, 8, 9, 10).

**Figure 11:**
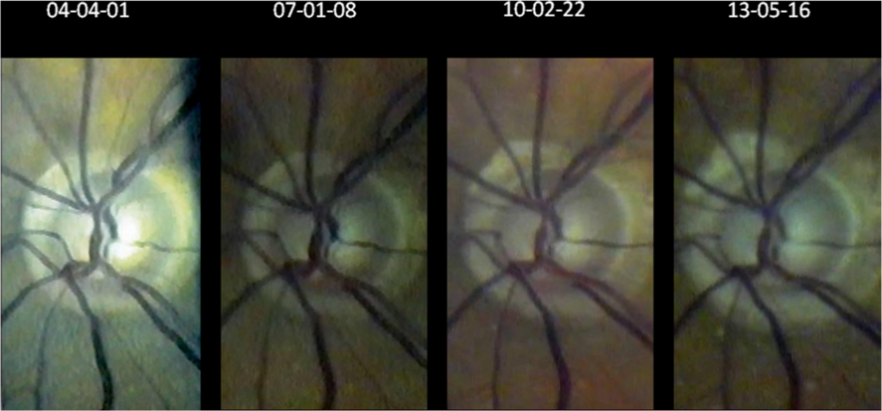
**Over 9 years progressing excavation of the right optic disc (seen through the slit lamp with fundoscopic lens +90D at 32x magnification) despite well-controlled intraocular pressure (for flicker test see Additional file** **1** **).**

### Image assessment

#### The flicker test

The flicker test is a precise method to detect changes occurring over time which is clearly superior to comparative evaluation of static images (see Figure 11). Fundus areas to be compared must be projected over one another, which we do manually using PowerPoint® for rotation and movement. We rarely need additional magnification - also possible with PowerPoint® - since we always try to use the same settings during an examination. By flicking between adjacent foils (pathological) alterations become evident as movement of structures (see Additional file 1). This is frequently not as obvious as eg. a new bleeding or progression in macular disease (Figure 10), but may be as subtle as e.g. a change in the course of retinal vessels. Individual retinal vessels in general serve as reliable landmark if there is e.g. loss of neuroretinal tissue as in glaucoma (Figure 11), tissue contraction as occurs with epiretinal membranes or swelling due to retinal edema.

## Discussion

In this manuscript we described, how the functionality of a video slit lamp for fundus imaging can be extended by using easily accessible additions. For a structured discussion the aforementioned pieces of equipment (converging lens, aperture) and image processing will be applied to slit lamp imaging of various parts of the fundus following the topographic order below. We use this opportunity to briefly mention and draw parallels to more advanced technology for retinal imaging as indicated. A more detailed comparison however, would exceed the purpose of this manuscript. For further reading on that issue we recommend (Gellrich 2014a).

1. posterior pole - fundus photography, FAG
2. optic nerve - HRT
3. macula - OCT, RTA, autofluorescence
4. fundus overviews - Optomap
5. peripheral fundus findings - Goldmann fundus contact lens
6. fundus perimetry - SLO.

### 1. The posterior pole

We consider any image that equally displays the optic nerve and macula, but not the peripheral retina, as a posterior pole image. This is the classic segment we have been familiar with for decades from standard 30-60° fundus photography (Liesenfeld 1959). Despite the beauty of displaying a large fundus section it should be kept in mind that by far most of the clinically important retinal pathology occurs within the temporal vessel arcades. For this fundus area we are now able to produce videographic images with the slit lamp which can be compared not only to non-mydriatic, but even to mydriatic techniques in fundus photography (see Figure 2):

A 10-15° fundus strip of the optic nerve and macula each (green light and 20x magnification through a +90D lens) is videographed by a slit lamp through a dilated pupil (>3 mm). Using the image processing program Hugin® one can meld these two images into a relatively homogeneous composite revealing approximately 15-25° of the fundus. This representation is not only appropriate for conditions like geographic atrophy (Figure 10), epiretinal membrane (Figure 2) and diseases such as vessel occlusion or diabetes. In comparison to our previous method showing these two images of the macula and the optic nerve only adjacent to each other without overlap (Gellrich 2011b) now not only a proper topographical reconstruction is possible, but also a more accurate clinical follow up can be performed with the flicker test (Figure 10).

White light has always been the preferred colour in classic fundus photography (Liesenfeld 1959; Littmann 1974) which is even older than the slit lamp itself (Dimmer 1907; Gerloff 1891; Jackman & Webster 1886). Even though no common standard of green light for the slit lamp exists we tend to prefer red free light for fundus videography, as it usually displays the fovea’s position more clearly (Gullstrand 1918) and epiretinal membranes (Vogt 1921) and smaller vessels with higher contrast (Affolter 1917; Vignal et al. 2007; Vogt 1925). Furthermore many signs of vascular disease like new vessel formation, microaneurysms, exudates and cotton wool spots (as indicators for retinal ischemia) can be seen so clearly with green light, that we rarely miss additional information which could be supplied by fluorescein angiography (Dithmar & Holz 2008). Although these facts are known principally for many years hardly any images captured with a slit lamp can be found neither presented at scientific meetings nor used in clinical practice.

### 2. Optic nerve

The slit lamp has always been used to identify risk factors for glaucoma (Vogt 1930, 1931, 1942). Recently we have shown that corneal thickness can also be estimated with the slit lamp (Gellrich 2012; Gellrich 2010; Koby 1930), which is important for correct interpretation of the IOP. Most important, however, in monitoring glaucoma is capturing the optic nerve’s morphology, which is still often summarized in a c/d ratio with considerable inter-individual variability. For a more accurate assessment we take an image of the optic disc and the circumpapillary area at high magnification (32x with + 90D lens) in green light (Figure 11). By flicking between images taken at different time points movement of the small vessels towards the edge of the cup reliably indicates loss of neuroretinal tissue within the optic disc (see Additional file 1). In Figure 11 we demonstrate a follow-up period for glaucoma with the slit lamp for more than 9 years - data not presented elsewhere. In our clinical records, however, meanwhile follow up periods of nearly 15 years are reached. The flicker test has previously been applied in principle to photographs of the optic nerve (Berger et al. 2000). There its accuracy has proven to resemble that of the HRT tomographic scanning method for glaucoma follow-up.

While the obvious clinical sign of a splinter hemorrhage may escape an HRT examination, HRT also provides volumetric figures which is not possible with the slit lamp (Kroll et al. 2008). The overall area of the optic disc, however, can be estimated from a slit beam with known dimensions projected on the fundus and the magnification of the fundus lens which is determined by its refractive power (0.75 for +90D, 1.15 for +60D and 1.67 for +40D) (Ansari-Shahrezaei et al. 2001). A more accurate method is to assess the overall magnification which occurs during transmission of the viewed image (through the slit lamp) onto the monitor screen. After calibrating the system with a linear scale held in front of the slit lamp objective distances and surface areas can be measured on the monitor or later on the .jpg file with the Datinf® program (Tübingen).

Ideally, the calculations mentioned above can only be applied if the light leaves a schematic eye at parallel beams, that is, if the patient is emmetropic. Quantitative relations, however, between lenses to compare are not affected if standard magnification values are applied to the same single eye, which we did for the dimensions given in Figure 2. To achieve exact retinal dimensions, however, the axial length of a given eye and its refractive status are required. If the patient is hyperopic the fundus appears at a little higher magnification (and less magnification in myopia) (Kaschke et al. 2014). This effect, however, is smaller the higher the power of the fundoscopic lens and the smaller the patient’s refractional error is. Furthermore, if in a hyperopic eye the distance between the corneal apex and the hand held lens is enlarged the fundus appears at a little lower magnification (higher magnification in myopic eye) (Rotter 1955).

For the start we recommend checking length and area calculations against optic-disc measurements taken by HRT (Dreher 1991) - keeping in mind that the main application of slit lamp measurements on the fundus is differentiating normal-sized from megalo- and micropapillae (Haustein et al. 2009).

### 3. Macula

Imaging the macula is becoming ever more important in practical clinical routine. While FAG assessment of the macula had been a standard procedure for decades, OCT with its capacity for depth analysis now plays a major role (Hee et al. 1995). In many modern textbooks on retinal imaging, even the new technique of autofluorescence (Schmitz-Valckenberg et al. 2008) is given more attention than is the slit lamp’s potential for assessing macular disease.

We find it helpful to examine the macula in a structured manner with increasing magnification (Gellrich 2013a):

- If CSC (central serous chorioretinopathy) is suspected a +90D lens at relatively low magnification (12x) can be used. Without stereoscopic vision (which is, of course the case in fundus videography with the camera attached to only one ocular) and without accompanying signs such as exudates or bleeding the oval macular light reflex should be observed: While this reflex vanishes in all people with increasing age it is often interrupted, sometimes even lost in the younger group affected usually by CSC (due to bulging of the retina from subretinal fluid). This is more obvious when compared to the other eye. Follow-up of the retinal surface contour in CSC with the slit lamp is, however, not easy, and it can never match the accuracy of OCT imaging of the subretinal space (Hee et al. 1995).
- For imaging of structures like epiretinal membranes or macular disease involving a larger area, we advise the setting described above for the posterior pole (see 1. Posterior pole). The flicker test will reveal changes in the course of individual vessels indicating membrane contraction. This test also makes changes during progressive geographic atrophy very obvious to both patient and examiner (Figure 10). For many practical ophthalmologists, this information should suffice to adequately manage the patient’s disease, and it remains questionable as to which cases benefit from the more refined method of autofluorescence in dry macular degeneration (Schmitz-Valckenberg et al. 2008). This overview of the posterior pole also suffices for imaging Junius Kuhnt changes (Figure 10) and the macular changes attributable to vessel occlusion or diabetes.
- If only the macula is the region of interest, we find the use of a 3.5 mm circular aperture helpful (with the fovea in the center - see Figure 3), as it reflects the macula’s structure better than the slit light usually applied with its rather vertical extension. (In the Haag Streit slit lamp there is greater variety of aperture sizes). By increasing the magnification at the slit lamp, there is always a loss of light intensity for the examiner, which plays a role in darkly pigmented eyes, in conditions like retinal vein occlusion, or with cataract; this may lead to inacceptable image degradation, particularly when green light has been used. We therefore find the combination of +60D lens (which itself offers 1.5-fold magnification compared to using a +90D lens) and 20x slit lamp magnification (see Figure 3) preferable to the combination +90D/32x slit lamp magnification. In this manner, bleedings, drusen, macular hole (Figure 3) and edemas caused by subretinal neovascularization can be effectively visualized.
  If the circular aperture is moved further - without changing the slit light’s width - a position is available offering three different areas: a (near) half circle from the 3.5 mm circular aperture, a dark intermediate zone, and a (near) semi-circle from the crescent-shaped aperture (see Figures 1 and 5). This “structured” aperture can be used to guide fixation (for example, ask the patient to look into the lower corner of the larger semi-circle and he will); but the setting can also reduce blinding (ask the patient to fixate on the dark intermediate area). These simple changes in the aperture system result in improvement for videographic evaluation by addressing two of the most important problems for patients in slit lamp biomicroscopy of the fundus: loss of orientation and blinding. Therefore slit lamp manufacturers should offer a professional solution rather than preserving an aperture system dating back to the early days of the slit lamp and entirely reflecting the needs of anterior segment biomicroscopy (see Figure 1).
- The last step in examining the macula is assessment of its surface contour. This is obviously advantageous in conditions such as macular hole, macular edema or drusen, as well as pigment epithelial detachment. Clinical binocular biomicroscopy usually offers a stereoscopic view of the macula, which can also be obtained (with effort) in stereophotography (Braley et al. 1970; Mártonyi et al. 2007). In videography, the camera is attached to one ocular, and we propose projecting e.g. a narrow vertical slit at a 5-15° angle through a +60D lens onto the fundus. It will be deflected to the side of the illumination arm in cases of fundus elevation, and away from it in a macular hole. We usually repeat this examination by swinging the illumination arm at the same angle to the other side. With experience, the “typical” shape of the projected slit is as follows: fuzzy edges (edema), smooth curve away from (fovea in young patients) or towards the incident light (drusen) and sharp, steep boarder between normal and diseased retina (pigment epithelial detachment (Gellrich 2014a), macular hole - see Figure 6). Sometimes, especially if the area to be assessed is confined to the fovea as in macular hole, we advise using higher magnifications (e.g. a +40D lens), but this usually requires a smaller angle (5°) between observation and illumination (see Figure 6). In comparison to the Watzke-Allen test, which relies on the patient’s interpretation what he is seeing, the procedure we suggest entirely depends on the skill and knowledge of the examiner.

Furthermore, by using standardized examination conditions (fixed angle between observation and illumination) the degree of deflection of the projected slit may be used to measure and follow-up surface height in the macular area with the slit lamp: In the example of Figure 6 we determined the depth of the macular hole by trigonometric functions as 0.55 mm) which is comparable to the OCT- measurement (0.44 mm). Interestingly there is a larger difference if we compare the vertical extensions: Which is 0.52 mm for the slit lamp and 1.12 mm for the OCT. A reason may be that for the slit lamp which only gives the “bird’s view” the real extension of the macular hole is hidden by its bulging edges (see Figure 6).

It has to be kept in mind that the slit lamp based evaluation allows analysis of the shape of the retinal surface contour only even though we see parallels between our slit lamp based procedure of measurement and the way an RTA (scanning retinal thickness analyzer) works (Zou et al. 2006). The RTA, however, images a green He-Ne laser slit beam (540 nm) projected obliquely onto the retina and retinal thickness is calculated from the separation between the reflections from the vitreoretinal and the chorioretinal interface.

OCT additionally gives information about intraretinal structure and the subretinal space (Konno et al. 2001). The impressive effects of OCT-documentation, however, are usually enhanced (approx. 4x) by different scales applied to the z-axis and to the x-/y-representation (see Figure 6).

The experienced clinician and examiner at the slit lamp, who is familiar with the application of more refined examination techniques of the fundus will be able to accompany by far most of his patients suffering from macular pathology without feeling the need to perform OCT-images. There will, however, be situations (e.g. early stages of macular hole with intraretinal cyst formation, follow up of central serous chorioretinopathy and diffuse macular edema), in which OCT-imaging eliminates diagnostic doubts. On the other hand, in advanced stages of macular diseases OCT on its own as a “fine tune” instrument frequently seems inappropriate to monitor the disease course (Figure 10).

While additional pathology as moderate cataract and synchisis scintillans does not affect much the quality of OCT-imaging, slit lamp videography earlier suffers from degradation of image quality. This effect is even stronger for “weak” fundus lenses (e.g. +20D lens) and hampers their clinical use at the extremely high magnifications (on our monitor up to 300x) they offer theoretically. Also their potential light toxicity (due to greater light intensity on a given fundus area) should be kept in mind, particularly when examining the fovea (Kohnen 2000). Considering all this from the examiner’s point of view who aims to adequately handle as many clinical situations at the slit lamp as possible an OCT extension of his instrument would be an ideal solution (Müller et al. 2010; Stehouwer et al. 2010).

### 4. Fundus overviews

There will, however, be occasions when larger parts of the fundus should be documented (Figure 8). This is the case in choroidal melanomas (Figure 7), but also in vitreoretinal surgery, occlusive vessel disease and diabetic retinopathy. Individual images may be taken at relatively low magnification (e.g. 8x or 12x) through a +90D lens to accommodate larger fundus areas. They may be stitched together digitally using the program Hugin®, which we already described in the section for posterior pole imaging, to create conclusive fundus documentation. Such software is available for fundus cameras but not so far for slit lamp imaging (Gellrich 2014a; Hackel 2005; Mody et al. 2000).

This technique may not be applied successfully if the clinical picture varies quickly with time (e.g. with bullous retinal detachment) as the program Hugin® requires setting fix points and no change in tissue structure as a basis for digital melding. Therefore if we only intend to indicate the involvement of a retinal area with a particular condition (e.g. in conjunction with vein occlusion or laser treatment), we still use our previous method of arranging representative areas in a scheme which devides the whole fundus in a topographic 3x3 pattern (Gellrich 2009b; Gellrich 2011c). Earlier on we calculated that this simplified “panretinal representation” by using a +90D lens at 8x magnification with a field diameter of approx 45° covers up to 47% of the retinal area (Gellrich 2011d). Obviously with our newer technique which has no limits concerning the number of individual fields this proportion can be significantly increased. Without the help, however, of a professional software which is specifically developed for slit lamp videography this will take too much time and certainly not reach the capacity of the 200°-optomap technique, which claims to visualize 82% of the retinal area (Sherman et al. 2007).

### 5. Peripheral fundus findings

Equatorial findings such as retinal tears are usually beyond the reach of fundus photography. The peripheral retina is widely known as the domain for the Goldmann fundus contact lens (Eisner 1973), but because of the small sector displayed we do not use it for image reconstructions rather than as a basis for fundus drawings (Lotmar 1975). This is possible, however, with those images taken through a +90D lens (e.g. at 8x or 12x magnification). We usually try to trace the pathological changes back to the optic disc and thereby display its topographical relation and relative size to it. Interestingly, we obtain conclusive images of findings in the upper and lower retina even with undilated pupils, i.e. in an example of pre-retinal snowballs in a patient with sarcoidosis (Figure 9). The more fundoscopy requires skill and flexibility on the side of the examiner the less we see the need for a fixed position of the hand held fundus lens as suggested elsewhere (Rotter 1955; Abraham 1988).

### 6. Fundus perimetry

Not only the examination of the macula would benefit from a more refined aperture system, also fundus perimetry with the slit lamp - obviously not a common application - would be much easier to carry out if the apertures were “substructured” with greater detail (see Figure 5): Offering different aperture shapes and not only uniformly “dark” around the illuminated fundus area would allow fundus orientation for the examiner while projecting single light spots on the retina in a controlled manner. Using LED-projection techniques as currently employed in modern beamers instead of the homogenous slit beam would open up a new world for fundus evaluation with the slit lamp. At least it would give the examiner the chance to diagnose hemianopia, define the borders of retinoschisis (Kroll et al. 2008) or assess whether a chorioretinal scar causes an arc scotoma following the direction of the crossing optic nerve fibres. Of course, fundus perimetry with the slit lamp can never be as precise as fundus perimetry using scanning laser ophthalmoscopy technique (Kaschke et al. 2014), but we find it helpful especially in handicapped patients where classical perimetry is not possible - even with our provisional solution using two half circles each being part of a “proper” aperture (see Figure 5).

## Conclusion

Although it is still regarded as the most indispensable instrument in clinical practice there is no slit lamp on the market that can exploit this instrument’s potential for fundus imaging. This article points out changes in the instrument itself, its examination equipment and the need of a specific videographic software (Gellrich 2011c). All the necessary items are available, but it is up to the manufacturer’s will to bring them together to an “all-in-one” practicable solution with the same reliability as we are used to it from our slit lamp in daily work. Moreover, the image resolution offered in this article represents the minimum of what is possible, because the camera we use and the videoprinter (for framegrabbing with storage capacities for single images around 100 kB) are more than ten years old - still providing us with disease follow ups over this whole period.

The spectrum of fundus applications with the slit lamp could be extensive: It spans from macular to glaucoma disease, from imaging large parts of the retina to peripheral anomalies, and in that regard it certainly exceeds the capacities of any other modern “high-end” instrument for retinal diagnostics. Such instruments certainly do offer measurement accuracy beyond that of the slit lamp and are designed that they can be used on the patient by non-specialists, but they always focus on specific parts of the eye (Kaschke et al. 2014). The slit lamp, working with visible light and largely dependent on skill and knowledge of its user, still has the capacity to image nearly any finding in ophthalmology (Gellrich 2009a), not only anterior segment (Meyner 1976; Sickenberger 2011), but also squint (Gellrich 2009e; Gellrich 2013b), fundus (Gellrich 2011d; Gellrich 2011e) and even matters of refractive surgery (Gellrich 2014b). If we want to continue using it as a basic and universal diagnostic tool - affordable for any ophthalmologist - we have to understand its principles, express what is clinically needed and demand what is feasible in order to make the manufacturers develop it further.

## Additional file

Additional file 1:
**(Same images as in Figure** 11 **) Here the reader himself can perform the flicker test to visualize progression of glaucomatous disc cupping: By flicking between adjacent foils (adjusted by PowerPoint®) the increase in peripapillary atrophy and movement of the small vessels towards the cup edges are obvious - particularly in the nasal part.** The flicker test is clearly more accurate to detect the changes than static comparison of images (see Figure 11).
