# Supplementary figures and images for: The fundus slit lamp
Source: Springerplus. 2015 Feb 3;4:56. doi: 10.1186/s40064-015-0838-5 (PMC4401483; doi:10.1186/s40064-015-0838-5)

## Slide 1
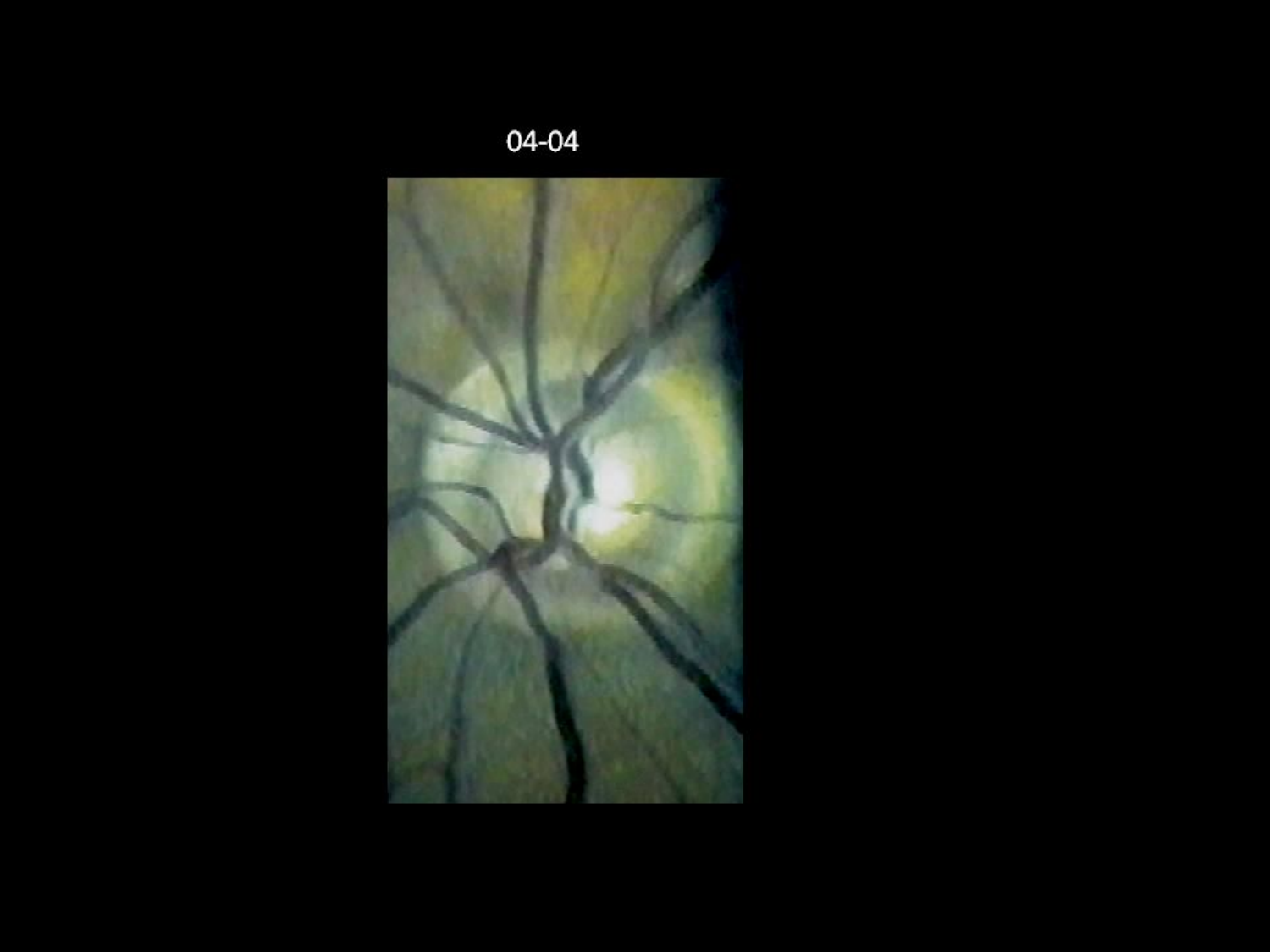

#

## Slide 2
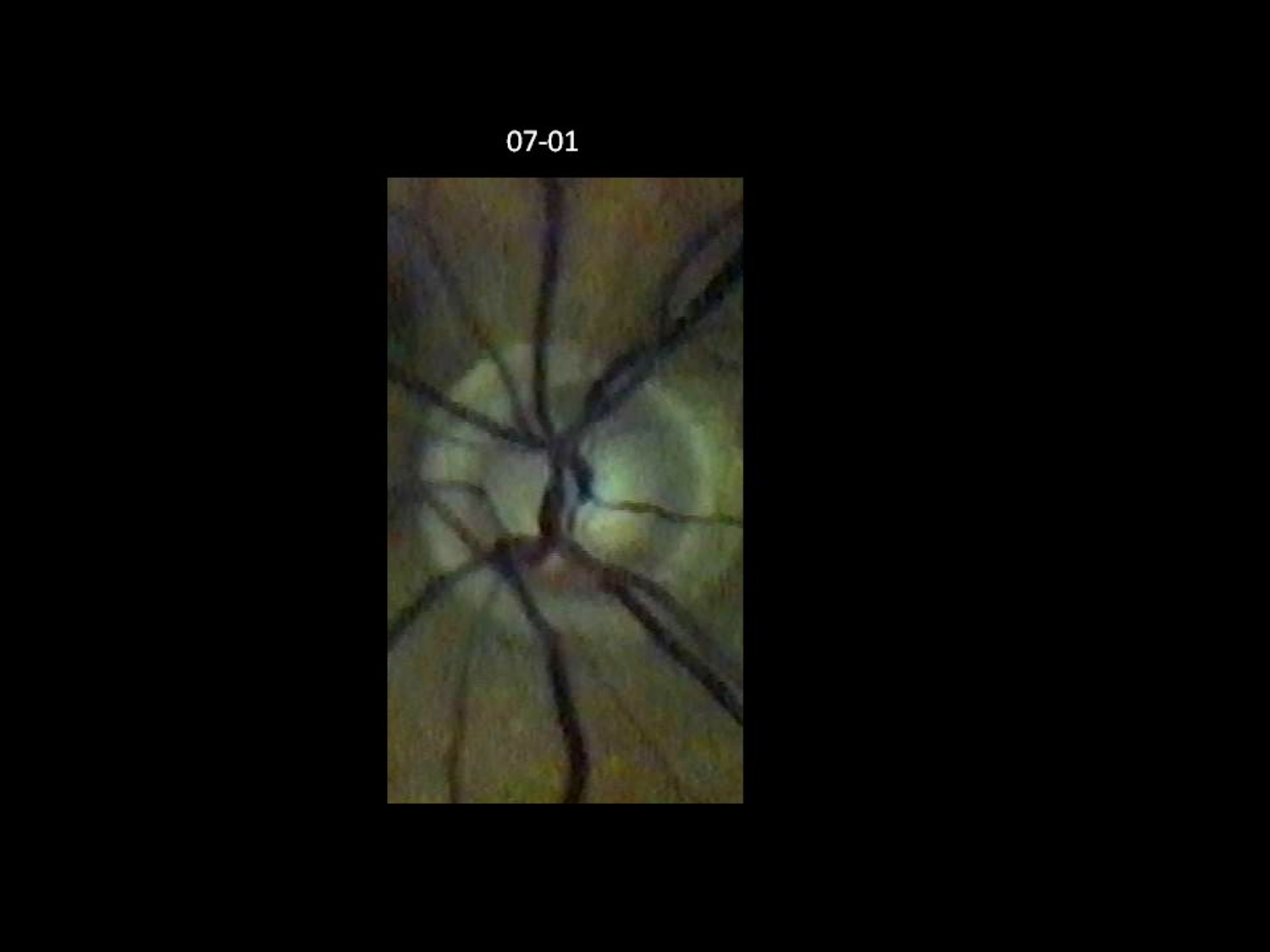

#

## Slide 3
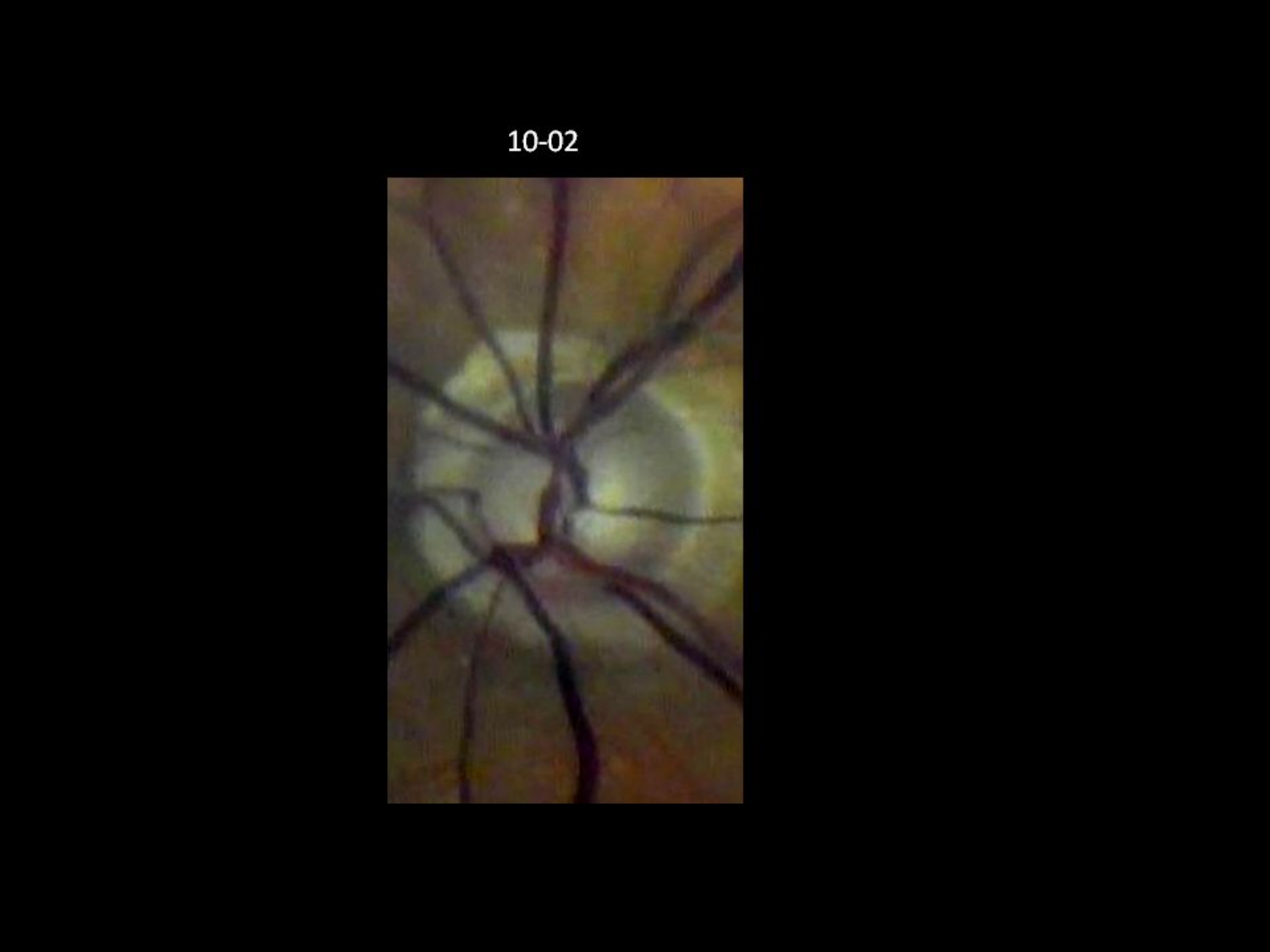

#

## Slide 4
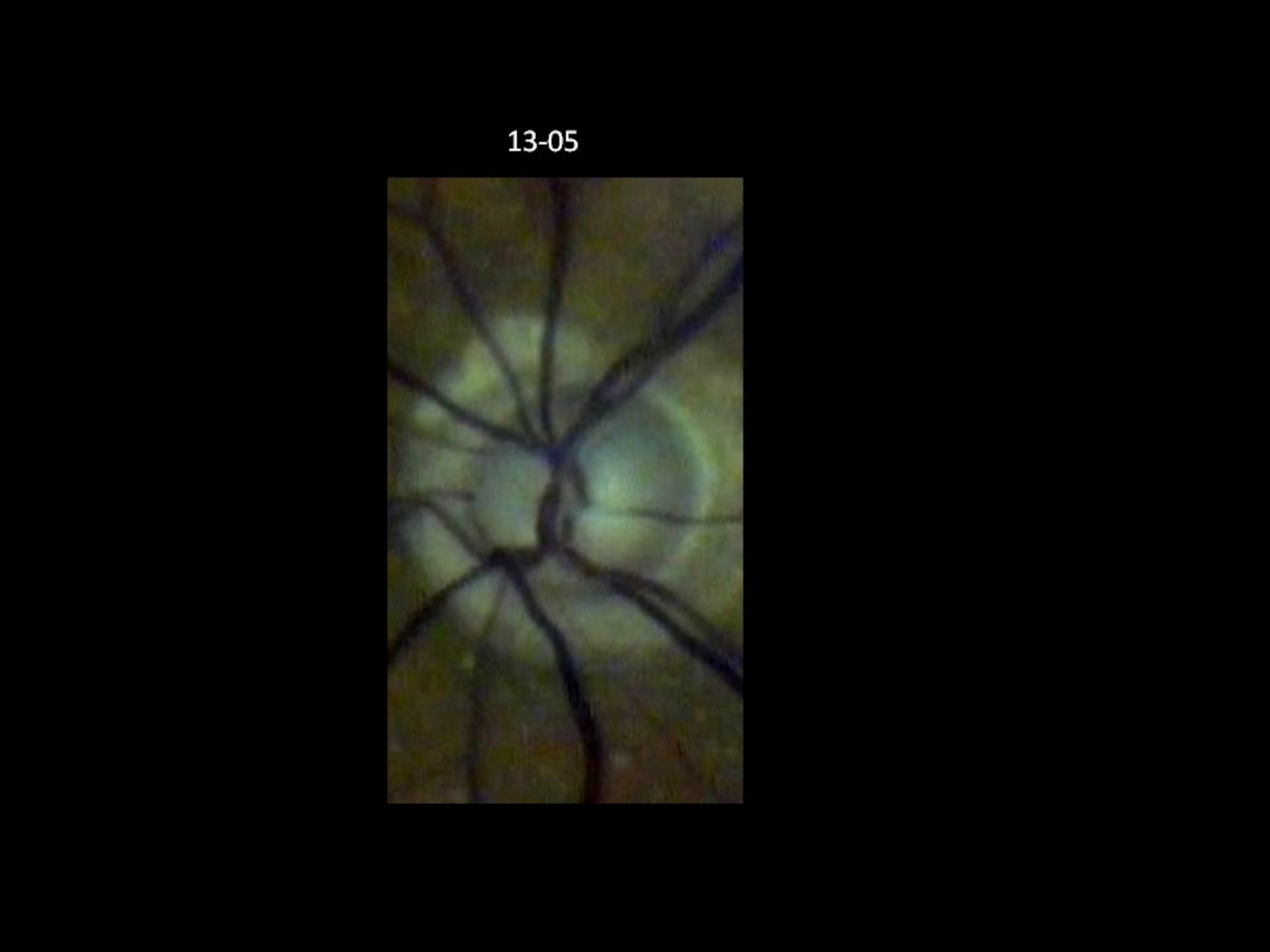

#

## Slide 5
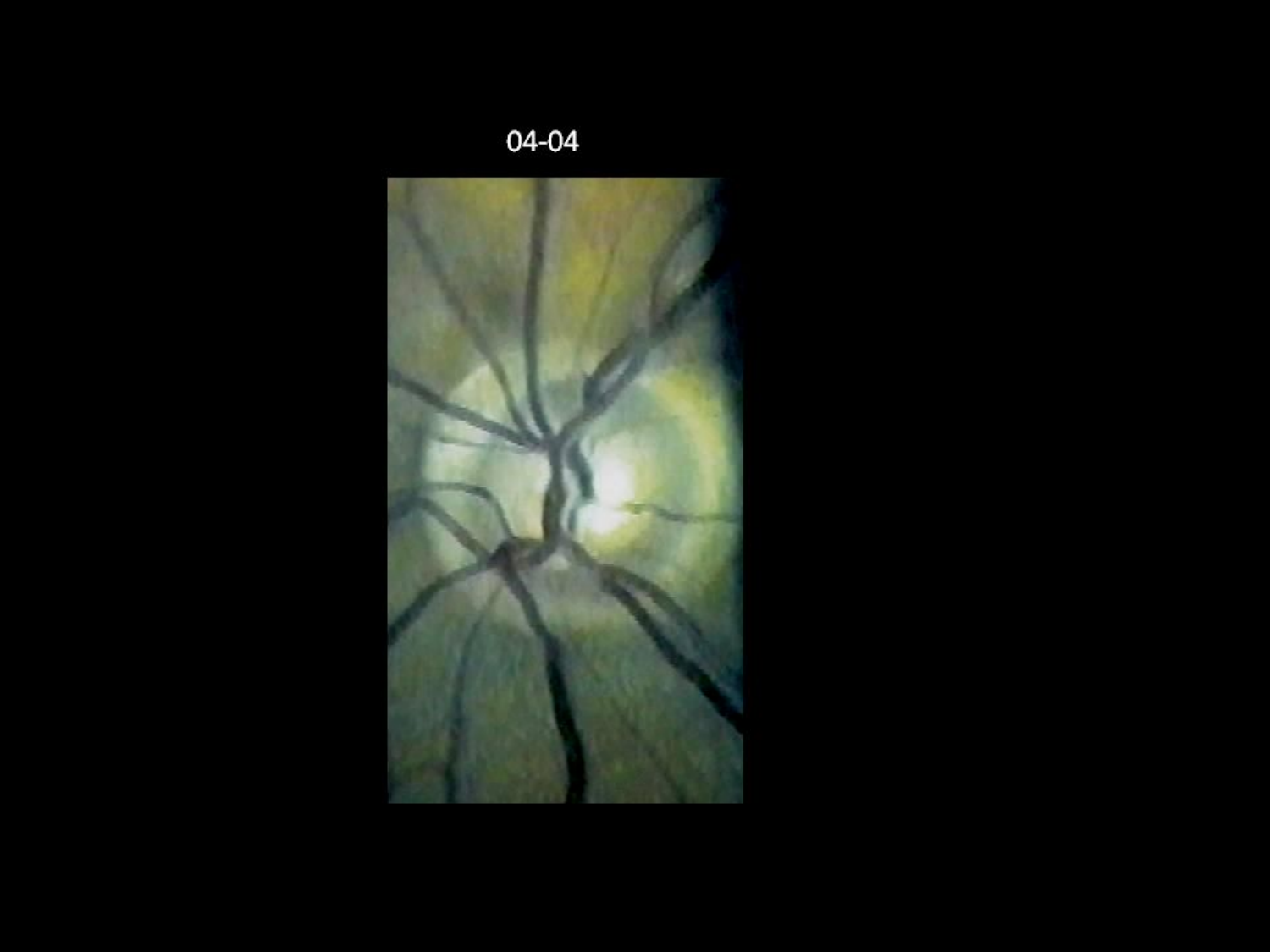

#

Supplement: Additional file 1: — (Same images as in Figure 11 ) Here the reader himself can perform the flicker test to visualize progression of glaucomatous disc cupping: By flicking between adjacent foils (adjusted by PowerPoint®) the increase in peripapillary atrophy and movement of the small vessels towards the cup edges are obvious - particularly in the nasal part. The flicker test is clearly more accurate to detect the changes than static comparison of images (see Figure 11). [file 40064_2015_838_MOESM1_ESM.pptx]
